# Analysis of the Resolution Rate of Complications in Obese Joint Replacement Patients

**DOI:** 10.5435/JAAOSGlobal-D-25-00079

**Published:** 2025-11-10

**Authors:** Austin Schwartz, Nicholas Brown, Michael Wesolowski

**Affiliations:** From the Loyola University Chicago Stritch School of Medicine (Dr. Schwartz); the Department of Orthopaedic Surgery and Rehabilitation, Loyola University Medical Center (Dr. Brown); and the Department of Statistics and Clinical Research, Loyola University Medical Center (Mr. Wesolowski), Maywood, IL.

## Abstract

**Introduction:**

Due to the high risk of complications, the body mass index (BMI) has been a commonly used cutoff metric for joint replacement surgery. However, the percentage of these complications that are treatable has been minimally reported on. This study seeks to quantify the type and incidence of complications that were treated and resolved within 90 days.

**Methods:**

The demographics, comorbidities, perioperative variables, and complications were reviewed for 700 patients with BMI >40. 475 patients underwent total knee replacement (TKAs) and 225 underwent total hip replacement (THAs). Univariable and multivariable hierarchically generalized linear mixed models (GLMMs) were utilized to control for relevant covariates.

**Results:**

211 of the total 700 patients had at least one complication. 205 procedures resulted in a medical complication (29.3%), 105 surgeries resulted in a surgical complication (15.0%), 97 procedures required reoperation (13.9%), and 104 procedures required readmission (14.9%). 149 of the 211 (70.7%) complications were treatable. Among hip replacements on patients with a BMI >40, BMI did not demonstrate a significant overall effect on any unadjusted (p=0.94) complication rate or adjusted analysis (p=0.66). Among knee replacements on patients with a BMI > 40, BMI did not demonstrate a significant overall effect on any unadjusted complication rate (p=0.95) or adjusted analyses (p=0.66). BMI stratification was performed (40-44.99, 45-49.99, and > 50), and no appreciable difference in complications, treatable or non-treatable, were observed.

**Conclusions:**

Although high complication rates were observed in this population rate with significant preoperative morbidities, the majority were treatable.

Obesity is the leading cause of preventable death worldwide, and it is associated with a higher incidence of medical comorbidities including cardiovascular, renal, osteoarthritis, and metabolic syndrome. It is well documented in the literature that the morbidly obese have a higher rate of postoperative complications. Infection rates are higher because of an impaired immune response, increased wound tension after closure, soft tissue edema, and poor microcirculation which collectively impair recovery. Obese patients are in a prothrombic and chronic state of inflammation leading to an increased rate of thromboembolism and stroke. In addition, the morbidly obese have decreased perioperative mobility which further increases the risk of thromboembolism and pulmonary issues.

Given the increased complication rate, many medical centers have instituted BMI cutoffs. On one hand, this is to protect patients from a medical complication. However, it is well documented that heavier patients with more complications add additional cost for the hospital. As illustrated by Daniel et al,^1^ many surgeons advocate for continuing to operate on heavier patients because they feel BMI is a crude measurement of health, and when controlled for other comorbidities, it is less predictive. Bagge et al^2^ noted obese patients achieve good improvements in Patient-reported Outcome Measures (PROM's) despite a lower preoperative score which demonstrates why surgeons will cite the increased quality of life and increase in PROM's that occur despite elevated BMI.

Across several large patient cohort studies, wound infection, wound dehiscence, deep vein thrombosis (DVT), and revision were the most common postoperative complications associated with an increased BMI. However, more life-threatening complications including myocardial infarction (MI), cerebrovascular accident (CVA), and acute kidney injury have not strongly correlated with an elevated BMI.^3-6^ Few studies exist in the literature exploring how common complications resolve in the obese cohort. The purpose of this study was to determine the risk acute complications after total hip or knee replacements and to identify the rate to which these complications were treatable. The hypothesis was that many of the complications would resolve within 90 days of surgery.

## Methods

In this retrospective study, the LUMC database was used after IRB approval was granted. This database rendered 700 patients with a BMI greater than 40 from the years 2005 to 2022 who underwent a primary total hip (THA) or total knee arthroplasty (TKA). BMI was calculated using the standard formula of weight in kilograms divided by the square of height (meters). We recorded demographic data including age, sex, race, ethnicity, and insurance type. Any patient extrapolated from the electronic medical record that did not have a height and/or weight or an extreme BMI due to reporting errors (<13, >66) was excluded (n = 5). An additional four patients were followed up for less than 90 days. None of these patients had an identifiable complication.

Patient comorbidities were all recorded which include a history of MI, congestive heart failure, peripheral vascular disease, CVAs, connective tissue disease, chronic pulmonary disease, liver disease, diabetes type 1, diabetes type 2, dementia, kidney disease, DVT, malignancy, smoking, American Society of Anesthesiologists score, and comorbidities included in the Charlson Comorbidity Index. Intraoperative statistics were also collected which included duration of surgery, blood loss, intraoperative medications, hospital length of stay, and location of discharge.

Complications within 90 days of procedure were recorded and divided into treatable versus irreversible (nontreatable) complications. Treatable complications included postoperative complications that were resolved within a 90-day window. These included but were not limited to urinary tract infections, wound dehiscence, superficial wound infection, shortness of breath, DVTs, and resolved prosthetic joint infections. Nontreatable included postoperative complications that were not resolved within a 90-day window and led to long-term functional consequences; a worsened state than the patient had before surgery. These included but were not limited to CVA's, MIs, PE's, mortality, prosthetic joint infections which were not resolved, or any complication failing to resolve in the 90-day window (Supplementary Table 5, http://links.lww.com/JG9/A452). The resolution or persistence of a complication was verified by chart review.

Statistics were reported to describe the sample both overall and stratified by type of arthroplasty at the surgery level. Univariable and multivariable hierarchically well-formulated generalized linear mixed models estimated, respectively, the unadjusted and adjusted effects of BMI and other independent predictors on the logit of treatable complications, untreatable complications, and any complications after total joint arthroplasty in separate models. The data were stratified by type of total joint arthroplasty so that knee replacements and hip replacements were analyzed separately. Random intercepts accounted for within-patient correlation. These models featured a residual, subject-specific, pseudolikelihood estimation method, a binary distribution, and a logit link. Kenward-Roger approximations corrected the denominator degrees of freedom, and an unstructured covariance structure was used for the G matrix. Odds ratio estimates were reported for each predictor effect, along with corresponding 95% confidence intervals (CI) and two-tailed *t*-test *P* values. Omnibus Type 3 F test *P* values were reported for the overall effects of polytomous predictors. Independent predictors were selected for inclusion in multivariable models based on conceptual considerations and significance in univariable models. Patients were considered to be on a strong anticoagulant medication if they were taking direct oral anticoagulants, warfarin, lovenox, or heparin on admission. Statistical analyses were completed using SAS Version 9.4. In addition, BMI stratification was also done (BMI [40 to 44.99, 45 to 49.99, and >50]) to assess for appreciable differences in medical, surgical, treatable, and nontreatable complications.

## Results

After exclusions, this analytic sample consisted of 700 unique total joint arthroplasty procedures performed on 592 unique patients having a BMI ≥ 40 between January 1, 2005 and December 1, 2022 at LUMC (Supplementary Table 1, http://links.lww.com/JG9/A448). Four hundred seventy-five (67.9%) were total knee replacements, and 225 (32.1%) were THAs. Overall, patients had a BMI ≥ 50 for 83 procedures (11.9%), a BMI of 45 to 49.99 for 178 procedures (25.4%), and a BMI of 40 to 44.99 for 439 procedures (62.7%). Four hundred eighty procedures were done on female patients (68.6%), and 220 procedures were done on male patients (31.4%). The average age at surgery was 61.1 years (SD, 9.1), the median procedure length was 114 minutes Interquartile range (IQR), 100 to 139), and the median length of stay was 4.0 days (IQR, 3.0 to 4.0). Two hundred five procedures resulted in a medical complication (29.3%), 105 procedures resulted in a surgical complication (15.0%), 97 procedures required revision surgery (13.9%), and 104 procedures required readmission (14.9%). One hundred forty-nine procedures resulted in a treatable complication (21.3%), 62 procedures resulted in an untreatable complication (8.9%), and 211 procedures had any complication overall (30.1%; Supplementary Table 2 http://links.lww.com/JG9/A449, Supplementary Table 3 http://links.lww.com/JG9/A450, and Supplementary Table 4, http://links.lww.com/JG9/A451).

One hundred thirty-eight of the 475 knee replacements resulted in a medical complication (29.1%), 68 resulted in a surgical complication (14.3%), 64 required revision surgery (13.5%), and 71 required readmission (15.0%). Overall, of the 144 total complications, 103 were treatable (71.5%). Among knee replacements on patients with a BMI > 40, BMI did not demonstrate a significant overall effect on any complication (treatable or untreatable) in unadjusted (*P* = 0.95) or adjusted analyses (*P* = 0.66, Supplementary Table 4, http://links.lww.com/JG9/A451).

Sixty-seven of the total 225 hip replacements resulted in a medical complication (29.8%), 37 resulted in a surgical complication (16.4%), 33 required revision surgery (14.7%), and 33 required readmission (14.7%). Overall, 46 of the total 67 complications resulted in a treatable complication (68.7%). Among hip replacements on patients with a BMI > 40, BMI did not demonstrate a significant overall effect on any complications (treatable or untreatable) unadjusted (*P* = 0.94) or adjusted analysis (*P* = 0.66).

Among knee replacements on patients with a BMI > 40, an increased length of surgery in both the univariate and multivariate results demonstrated a significant effect on the complication rate (*P* = <0.01; OR: 1.62; 95% CI: 1.15, 2.27). In patients on anticoagulant therapy undergoing TKAs with a BMI > 40, a significant effect in both the univariate and multivariate analyses was also observed (*P* = 0.01; OR: 1.86; 95% CI: 1.19, 2.91). The odds of a treatable complication increased significantly per each three-unit increase in Charlson Comorbidity Index (*P* = 0.02; OR: 1.50; 95% CI: 1.07, 2.12). This effect remained significant and essentially unchanged after multivariable adjustment (OR: 1.55; 95% CI: 1.06, 2.27; *P* = 0.02).

In addition, BMI stratification was done (40 to 44.99, 45 to 49.99, and >50), and no significant difference in complications for hip or knee replacements, treatable or nontreatable, was observed (Supplementary Table 2, http://links.lww.com/JG9/A449 and Supplementary Table 3, http://links.lww.com/JG9/A450). Complication type was sorted perioperatively and during the 90-day postoperative period (Supplementary Table 5, http://links.lww.com/JG9/A452).

## Discussion

BMI cutoffs are a widely used criterion restricting TKAs and THAs in healthcare centers globally. Although evidence in the literature suggests a higher BMI is associated with more postoperative complications, few studies describe the percentage of short-term complications that can be treated and resolved. The analytic sample of 700 patients with a BMI > 40 over a 17-year span through univariable and multivariable generalized linear mixed models concluded most of the complications (70.7%) were treatable. Although this cohort demonstrated an increased risk of complications with a BMI > 40 (29.3%) and an increased risk of revision surgery (13.9%), most of the complications (70.7%) were treated and resolved within a 90-day span following the procedure.

The literature has also explored whether patients with an increased BMI have a higher rate of complications. Michalka et al^7^ noted in 191 patients undergoing THAs that although surgeons perceived that the arthroplasties in obese patients were more difficult, it did not translate to an increased risk of complications, surgery length, blood loss, or suboptimal implant placement. Ma et al noted in 2969 patients undergoing TKA that the overall complication rates in the early postoperative period were highest in the underweight and normal weight cohort and lowest in the overweight cohort. Early postoperative complications were defined as complications occurring during the hospital stay.^8^ These results supported the U-shaped relationship that exists between BMI and readmission and revision surgery due to complications. George et al^9^ highlighted in a study sample of 150,934 TKAs that considerable variations existed between BMI and complication rates regarding outcomes including readmission and revision surgery. In data from 12,355 patients undergoing THA or TKA with a BMI > 40, Friedman et al noted there were no notable differences in the rate of DVTs, Pulmonary Embolism (PEs), or bleeding. The only statistically notable differences found when compared with a BMI cohort < 40 was increased risk of erythema, peripheral edema, diarrhea, abdominal pain, wound inflammation/infection, and respiratory tract/lung infection.^10^ Although there seems to be a consensus that there is an increased risk of complications in the obese, some evidence in the literature seems to conflict that sentiment, particularly when the data are controlled for other patient demographics and comorbidities. In addition, evidence in the literature states that obese patients have equal if not greater improvements in quality of life as compared with nonobese patients. This was explored by McCalden et al, who found in 3,290 patients undergoing THA, large increases in Western Ontario and McMaster Universities Osteoarthritis Index, Harris Hip Score, and SF-12 indexes suggested an equal if not greater improvement compared with nonobese patients.^11^

As previously discussed, the BMI cutoff criterion can negatively affect patient quality of life. Giori et al^12^ highlighted strong evidence toward the negative impact BMI cutoffs can have on the quality of life in patients dealing with progressive Osteoarthritis (OA). In a 3-year study reviewing more than 27,000 joint arthroplasties, Giori demonstrated how many patients would have been outright denied access to a complication-free procedure if the hospital had been using a BMI > 40 cutoff criterion. 90.8% of procedures would have proceeded without complications, and more interestingly, if a BMI cutoff criterion of 30 was used, the eligibility criteria was almost identical to flipping a coin. DeMik et al^13^ noted that although lowering BMI cutoffs resulted in fewer complications, it consequentially limited access to complication-free THA. In 192,394 patients undergoing THA, DeMik demonstrated that although using BMI cutoffs of 45 or 50 increased surgical time and American Society of Anesthesiologist scores, the higher cutoffs allowed for a similar proportion of patients to undergo complication-free THA and resulted in fewer patients being denied complication-free procedures. If a BMI cutoff of 35 was used, only 75.9% of complication-free procedures proceeded compared with the 95.2% who would not experience a complication at a BMI cutoff of 50. Wang et al^14^ noted the BMI cutoff criterion may worsen racial and socioeconomic disparities. The study exemplified payment structures and clinical-decision making algorithms set inflexible cutoffs that disproportionately discourage performing lower extremity arthroplasty on non-Hispanic Blacks and individuals of lower socioeconomic status.

Although there is a reasonable stance that obesity is a reversible comorbidity, dedicated weight clinics as well as multidisciplinary services and bariatric surgery often fail to produce notable weight loss. Ricciardi et al^15^ demonstrated that only 8% of patients denied surgery due to BMI cutoff criterion eventually reached the criterion parameters.^15^ Without a reliable pathway for weight loss, this places a cohort with a nearly irreversible comorbidity in a predicament. In addition, weight reduction before surgery may not reduce risk as noted by Inacio et al.^16^ By contrast, Keeney et al^17^ showed that even a modest 20-pound weight loss improved outcomes. Hence, even if a patient cannot reach the BMI 40 threshold, encouraging weight reduction may improve outcomes.

There is also an argument to be made that BMI may not be the most effective metric as a predictor for unfavorable acute perioperative outcomes. BMI has been demonstrated to have a good correlation with body fat percentage, yet the accuracy of BMI to diagnose obesity is limited. It is particularly limited in BMI ranges >30 where the metric underrepresents more than half of people with excess fat.^18^ Furthermore, in a publication reviewing 539,737 patients, metabolic syndrome was demonstrated to have a more predictive effect in high-risk patients who underwent TKA or THA then using BMI alone as the crude measurement. Metabolic syndrome was calculated using a set of defining components including hypertension, diabetes, and obesity.^19^ This study further supported evidence of moving away from a strict BMI cutoff alone in determining eligibility. If the objective of BMI cutoff criterion is to identify the unhealthy to prevent complications, a more accurate metric should be considered. Humphreys^20^ identified certain races may have a higher proportion of fat mass for a given BMI than White patients, while the reverse is true for most Black patients and Polynesian.

Limitations of this study include the somewhat subjective nature of treatable versus nontreatable complication. This study considered complications resolved within a 90-day time span without long-term functional consequences a treatable complication. Nontreatable complications were not resolved and led to long-term functional consequences. It can be argued that a prosthetic joint infection, even if quickly treated and resolved leading to vastly improved functionality, would be considered a severe complication. In addition, complications including prosthetic loosening, dislocation, late infection, or fracture that can occur months to years after surgery were not explored which underscores that complications beyond the 90-day postoperative window were not explored. Furthermore, many guidelines for BMI cutoffs have been established because of difficulty of the surgical procedure, accuracy of implant placement, and prosthetic failure rates which were not explored in this study aside from 90-day complications. In addition, we do not have patient-reported outcome data comparing those who had a resolved complication with those without. Therapeutic anticoagulant medication was grouped as a whole, so individual anticoagulant effects on complication rates were not explored. In addition, a cohort comparison in patients with patients with a BMI under 40 was not done.

Despite being an incomplete measure of health and predictor of complications, most studies have demonstrated an association between increased BMI and complications. However, the BMI cutoff criterion may prevent access to total hip or knee arthroplasty and worsen the quality of life of many patients with a BMI > 40 possess. In addition, other metrics may provide a more accurate prediction of acute perioperative outcomes and will not inaccurately deny access to care. Nevertheless, this study did demonstrate a very high rate of complications and revision surgeries in this obese patient population. However, most of these short-term complications were treatable (70.7%). Surgeons may benefit from using these data to discuss the risks and benefits with their future patients.

## Supplementary Material

SUPPLEMENTARY MATERIAL
