# Supplementary material for: Analysis of the Resolution Rate of Complications in Obese Joint Replacement Patients
Source: J Am Acad Orthop Surg Glob Res Rev. 2025 Nov 10;9(11):e25.00079. doi: 10.5435/JAAOSGlobal-D-25-00079 (PMC12604657; doi:10.5435/JAAOSGlobal-D-25-00079)
Supplement: SUPPLEMENTARY MATERIAL [file jagrr-9-e25.00079-s001.docx]

JAAOS table 1

JAAOS Supplemental Data

Supplemental Table 1: Patient Demographics, Comorbidities, & Post-op Complications by Type of Arthroplasty

| **Variable** | **Total Knee Arthroplasty**  **(n = 475)** | | **Total Hip Arthroplasty**  **(n = 225)** | | **Overall**  **(n = 700)** | |
| --- | --- | --- | --- | --- | --- | --- |
| BMI |  | |  | |  | |
| > 50 | 59 (12.42) | | 24 (10.67) | | 83 (11.86) | |
| 45 – 49.99 | 124 (26.11) | | 54 (24.00) | | 178 (25.43) | |
| 40 – 44.99 | 292 (61.47) | | 147 (65.33) | | 439 (62.71) | |
| Sex |  | |  | |  | |
| Female | 346 (72.84) | | 134 (59.56) | | 480 (68.57) | |
| Male | 129 (27.16) | | 91 (40.44) | | 220 (31.43) | |
| Race |  | |  | |  | |
| Other | 28 (5.89) | | 8 (3.56) | | 36 (5.14) | |
| Black/African American | 109 (22.95) | | 41 (18.22) | | 150 (21.43) | |
| White | 338 (71.16) | | 176 (78.22) | | 514 (73.43) | |
| Ethnicity |  | |  | |  | |
| Missing | 4 (0.84) | | 3 (1.33) | | 7 (1.00) | |
| Hispanic/Latino | 47 (9.89) | | 12 (5.33) | | 59 (8.43) | |
| Not Hispanic/Latino | 424 (89.26) | | 210 (93.33) | | 634 (90.57) | |
| Insurance |  | |  | |  | |
| Uninsured | 1 (0.21) | | 0 (0.00) | | 1 (0.14) | |
| Other | 11 (2.32) | | 3 (1.33) | | 14 (2.00) | |
| Self-Pay | 1 (0.21) | | 0 (0.00) | | 1 (0.14) | |
| Private | 222 (46.74) | | 102 (45.33) | | 324 (46.29) | |
| Medicare | 162 (34.11) | | 85 (37.78) | | 247 (35.29) | |
| Medicaid | 78 (16.42) | | 35 (15.56) | | 113 (16.14) | |
| Smoking |  | |  | |  | |
| Current | 30 (6.32) | | 22 (9.78) | | 52 (7.43) | |
| Former | 132 (27.79) | | 68 (30.22) | | 200 (28.57) | |
| Never | 313 (65.89) | | 135 (60.00) | | 448 (64.00) | |
| Diabetes | 150 (31.58) | | 74 (32.89) | | 224 (32.00) | |
| Strong Anticoagulant Medication | 266 (56.00) | | 116 (51.56) | | 382 (54.57) | |
| ASA Class |  | |  | |  | |
| Missing | 35 (7.37) | | 18 (8.00) | | 53 (7.57) | |
| 1 | 0 (0.00) | | 1 (0.44) | | 1 (0.14) | |
| 2 | 56 (11.79) | | 29 (12.89) | | 85 (12.14) | |
| 3 | 197 (41.47) | | 75 (33.33) | | 272 (38.86) | |
| 4 | 187 (39.37) | | 102 (45.33) | | 289 (41.29) | |
| Discharge Disposition |  | |  | |  | |
| Other | 177 (37.26) | | 96 (42.67) | | 273 (39.00) | |
| Rehab | 65 (13.68) | | 29 (12.89) | | 94 (13.43) | |
| LTAC | 91 (19.16) | | 31 (13.78) | | 122 (17.43) | |
| STAC | 3 (0.63) | | 0 (0.00) | | 3 (0.43) | |
| Home | 139 (29.26) | | 69 (30.67) | | 208 (29.71) | |
| Medical Complication | 138 (29.05) | | 67 (29.78) | | 205 (29.29) | |
| Surgical Complication | 68 (14.32) | | 37 (16.44) | | 105 (15.00) | |
| Require Reoperation | 64 (13.47) | | 33 (14.67) | | 97 (13.86) | |
| Require Readmission | 71 (14.95) | | 33 (14.67) | | 104 (14.86) | |
| Treatable Complication | 103 (21.68) | | 46 (20.44) | | 149 (21.29) | |
| Untreatable Complication | 41 (8.63) | | 21 (9.33) | | 62 (8.86) | |
| Any Complication | 144 (30.32) | | 67 (29.78) | | 211 (30.14) | |
| **Variable** | **n** | **Mean (SD)** | **n** | **Mean (SD)** | **n** | **Mean (SD)** |
| Age at Surgery (years) | 475 | 61.58 (8.51) | 225 | 60.16 (10.14) | 700 | 61.12 (9.08) |
| **Variable** | **n** | **Median (IQR)** | **n** | **Median (IQR)** | **n** | **Median (IQR)** |
| Charlson Comorbidity Index | 475 | 3.00 (2.00 – 4.00) | 225 | 2.00 (1.00 – 4.00) | 700 | 3.00 (2.00 – 4.00) |
| Length of Surgery (minutes) | 410 | 113.00 (98.00 – 137.00) | 198 | 121.50 (103.00 – 140.00) | 608 | 114.00 (100.00 – 139.00) |
| Length of Stay (days) | 475 | 4.00 (3.00 – 5.00) | 225 | 4.00 (3.00 – 4.00) | 700 | 4.00 (3.00 – 4.00) |
