# Supplementary material for: Analysis of the Resolution Rate of Complications in Obese Joint Replacement Patients
Source: J Am Acad Orthop Surg Glob Res Rev. 2025 Nov 10;9(11):e25.00079. doi: 10.5435/JAAOSGlobal-D-25-00079 (PMC12604657; doi:10.5435/JAAOSGlobal-D-25-00079)
Supplement: SUPPLEMENTARY MATERIAL [file jagrr-9-e25.00079-s002.docx]

JAAOS Table 2

Supplemental Table 2: Estimated Effects of BMI & Other Predictors on Treatable Complications Following Total Joint Arthroplasty, Stratified by Joint

| **Independent Variable** | **Total Knee Arthroplasty** | | | | | **Total Hip Arthroplasty** | | | | | | |
| --- | --- | --- | --- | --- | --- | --- | --- | --- | --- | --- | --- | --- |
|  | ***Unadjusted*** | | | ***Adjusted (n = 406)*** | | ***Unadjusted*** | | | | ***Adjusted (n = 198)*** | | |
|  | **n** | **OR (95% CI)** | **p** | **OR (95% CI)** | **p** | **n** | **OR (95% CI)** | **p** | **OR (95% CI)** | | **p** |  |
| BMI | 475 |  | 0.43† |  | 0.55† | 225 |  | 0.71† |  | | 0.60† |  |
| > 50 |  | 1.18 (0.58, 2.39) | 0.64 | 1.16 (0.51, 2.63) | 0.73 |  | 0.90 (0.28, 2.87) | 0.85 | 0.46 (0.10, 2.11) | | 0.32 |  |
| 45 – 49.99 |  | 0.73 (0.41, 1.29) | 0.28 | 0.74 (0.40, 1.40) | 0.36 |  | 0.69 (0.29, 1.66) | 0.41 | 0.90 (0.34, 2.34) | | 0.82 |  |
| 40 – 44.99 |  | 1.00 (REF) | - | 1.00 (REF) | - |  | 1.00 (REF) | - | 1.00 (REF) | | - |  |
| Sex | 475 |  |  |  |  | 225 |  |  |  | |  |  |
| Female |  | 1.14 (0.66, 1.94) | 0.64 | 1.21 (0.66, 2.23) | 0.54 |  | 1.53 (0.73, 3.21) | 0.26 | 1.25 (0.55, 2.86) | | 0.59 |  |
| Male |  | 1.00 (REF) | - | 1.00 (REF) | - |  | 1.00 (REF) | - | 1.00 (REF) | | - |  |
| Race | 475 |  | 0.63† |  | 0.24† | 225 |  | 0.34† |  | | - |  |
| Other |  | 0.92 (0.33, 2.53) | 0.87 | 3.50 (0.74, 16.45) | 0.11 |  | 0.50 (0.05, 4.66) | 0.54 | - | | - |  |
| Black/African American |  | 0.75 (0.42, 1.35) | 0.34 | 0.84 (0.42, 1.69) | 0.63 |  | 0.47 (0.16, 1.38) | 0.17 | - | | - |  |
| White |  | 1.00 (REF) | - | 1.00 (REF) | - |  | 1.00 (REF) | - | 1.00 (REF) | | - |  |
| Ethnicity | 471 |  |  |  |  | 222 |  |  |  | |  |  |
| Hispanic/Latino |  | 0.50 (0.19, 1.27) | 0.14 | 0.30 (0.07, 1.23) | 0.09 |  | 1.93 (0.48, 7.72) | 0.35 | - | | - |  |
| Not Hispanic/Latino |  | 1.00 (REF) | - | 1.00 (REF) | - |  | 1.00 (REF) | - | 1.00 (REF) | | - |  |
| Smoking | 475 |  | 0.78† |  | - | 225 |  | 0.36† |  | | - |  |
| Current |  | 0.70 (0.24, 1.99) | 0.50 | - | - |  | 1.42 (0.43, 4.69) | 0.56 | - | | - |  |
| Former |  | 0.93 (0.54, 1.58) | 0.78 | - | - |  | 1.73 (0.81, 3.73) | 0.16 | - | | - |  |
| Never |  | 1.00 (REF) | - | 1.00 (REF) | - |  | 1.00 (REF) | - | 1.00 (REF) | | - |  |
| Diabetes | 475 |  |  |  |  | 225 |  |  |  | |  |  |
| Yes |  | 1.00 (0.61, 1.64) | 0.99 | - | - |  | 0.67 (0.31, 1.45) | 0.30 | - | | - |  |
| No |  | 1.00 (REF) | - | 1.00 (REF) | - |  | 1.00 (REF) | - | 1.00 (REF) | | - |  |
| Strong Anticoagulant Medication | 475 |  |  |  |  | 225 |  |  |  | |  |  |
| Yes |  | 1.84 (1.13, 3.01) | 0.01* | 2.19 (1.24, 3.86) | 0.01* |  | 1.54 (0.76, 3.13) | 0.23 | - | | - |  |
| No |  | 1.00 (REF) | - | 1.00 (REF) | - |  | 1.00 (REF) | - | 1.00 (REF) | | - |  |
| Age at Surgery, 5-year increase | 475 | 1.00 (0.87, 1.15) | 0.98 | 0.92 (0.77, 1.11) | 0.40 | 225 | 1.10 (0.92, 1.31) | 0.31 | - | | - |  |
| Charlson Comorbidity Index,  3-unit increase | 475 | 1.22 (0.94, 1.58) | 0.14 | 1.26 (0.91, 1.75) | 0.17 | 225 | 1.50 (1.07, 2.12) | 0.02* | 1.55 (1.06, 2.27) | | 0.02* |  |
| Length of Surgery,  60-minute increase | 410 | 1.10 (0.79, 1.51) | 0.58 | 1.00 (0.69, 1.44) | 0.98 | 198 | 1.49 (0.90, 2.46) | 0.12 | 1.76 (1.01, 3.05) | | 0.04* |  |

*Significant at α = 0.05 level

†Type 3 omnibus p-value for overall polytomous predictor effect

CI = Confidence Interval; OR = Odds Ratio
