# Supplementary material for: Analysis of the Resolution Rate of Complications in Obese Joint Replacement Patients
Source: J Am Acad Orthop Surg Glob Res Rev. 2025 Nov 10;9(11):e25.00079. doi: 10.5435/JAAOSGlobal-D-25-00079 (PMC12604657; doi:10.5435/JAAOSGlobal-D-25-00079)
Supplement: SUPPLEMENTARY MATERIAL [file jagrr-9-e25.00079-s003.docx]

JAAOS table 3

Complications Following Total Joint Arthroplasty, Stratified by Joint

| **Independent Variable** | **Total Knee Arthroplasty** | | | | | | | **Total Hip Arthroplasty** | | | | | | |
| --- | --- | --- | --- | --- | --- | --- | --- | --- | --- | --- | --- | --- | --- | --- |
|  | ***Unadjusted*** | | | | ***Adjusted (n = 410)*** | | | ***Unadjusted*** | | | | ***Adjusted (n = 198)*** | | |
|  | **n** | **OR (95% CI)** | **p** | **OR (95% CI)** | | **p** | **n** | | **OR (95% CI)** | **p** | **OR (95% CI)** | | **p** |  |
| BMI | 475 |  | 0.33† |  | | 0.50† | 225 | |  | 0.46† |  | | 0.41† |  |
| > 50 |  | 0.58 (0.16, 2.15) | 0.41 | 0.38 (0.07, 1.93) | | 0.24 |  | | 1.79 (0.44, 7.32) | 0.42 | 1.38 (0.25, 7.71) | | 0.71 |  |
| 45 – 49.99 |  | 1.48 (0.70, 3.12) | 0.30 | 0.99 (0.41, 2.43) | | 0.99 |  | | 1.81 (0.64, 5.14) | 0.26 | 2.12 (0.70, 6.43) | | 0.18 |  |
| 40 – 44.99 |  | 1.00 (REF) | - | 1.00 (REF) | | - |  | | 1.00 (REF) | - | 1.00 (REF) | | - |  |
| Sex | 475 |  |  |  | |  | 225 | |  |  |  | |  |  |
| Female |  | 0.57 (0.28, 1.17) | 0.13 | 0.71 (0.31, 1.62) | | 0.41 |  | | 2.32 (0.80, 6.77) | 0.12 | - | | - |  |
| Male |  | 1.00 (REF) | - | 1.00 (REF) | | - |  | | 1.00 (REF) | - | 1.00 (REF) | | - |  |
| Race | 475 |  | 0.96† |  | | - | 225 | |  | - |  | | - |  |
| Other |  | 0.82 (0.17, 3.94) | 0.81 | - | | - |  | | - | - | - | | - |  |
| Black/African American |  | 0.92 (0.40, 2.11) | 0.85 | - | | - |  | | - | - | - | | - |  |
| White |  | 1.00 (REF) | - | 1.00 (REF) | | - |  | | 1.00 (REF) | - | 1.00 (REF) | | - |  |
| Ethnicity | 471 |  |  |  | |  | 222 | |  |  |  | |  |  |
| Hispanic/Latino |  | 1.00 (0.32, 3.17) | 0.99 | - | | - |  | | - | - | - | | - |  |
| Not Hispanic/Latino |  | 1.00 (REF) | - | 1.00 (REF) | | - |  | | 1.00 (REF) | - | 1.00 (REF) | | - |  |
| Smoking | 475 |  | 0.59† |  | | - | 225 | |  | - |  | | - |  |
| Current |  | 0.37 (0.05, 2.99) | 0.35 | - | | - |  | | - | - | - | | - |  |
| Former |  | 1.13 (0.54, 2.37) | 0.74 | - | | - |  | | - | - | - | | - |  |
| Never |  | 1.00 (REF) | - | 1.00 (REF) | | - |  | | 1.00 (REF) | - | 1.00 (REF) | | - |  |
| Diabetes | 475 |  |  |  | |  | 225 | |  |  |  | |  |  |
| Yes |  | 1.03 (0.50, 2.13) | 0.93 | - | | - |  | | 1.31 (0.50, 3.43) | 0.57 | - | | - |  |
| No |  | 1.00 (REF) | - | 1.00 (REF) | | - |  | | 1.00 (REF) | - | 1.00 (REF) | | - |  |
| Strong Anticoagulant Medication | 475 |  |  |  | |  | 225 | |  |  |  | |  |  |
| Yes |  | 1.39 (0.69, 2.79) | 0.36 | - | | - |  | | 1.06 (0.42, 2.69) | 0.90 | - | | - |  |
| No |  | 1.00 (REF) | - | 1.00 (REF) | | - |  | | 1.00 (REF) | - | 1.00 (REF) | | - |  |
| Age at Surgery, 5-year increase | 475 | 0.91 (0.75, 1.11) | 0.37 | - | | - | 225 | | 0.84 (0.68, 1.05) | 0.13 | - | | - |  |
| Charlson Comorbidity Index,  3-unit increase | 475 | 1.16 (0.80, 1.68) | 0.44 | 1.31 (0.88, 1.96) | | 0.18 | 225 | | 0.84 (0.48, 1.46) | 0.53 | - | | - |  |
| Length of Surgery,  60-minute increase | 410 | 2.07 (1.30, 3.30) | < 0.01* | 2.13 (1.30, 3.49) | | < 0.01* | 198 | | 1.42 (0.77, 2.64) | 0.26 | 1.35 (0.70, 2.59) | | 0.37 |  |

*Significant at α = 0.05 level

†Type 3 omnibus p-value for overall polytomous predictor effect

CI = Confidence Interval; OR = Odds Ratio
