# Supplementary material for: Analysis of the Resolution Rate of Complications in Obese Joint Replacement Patients
Source: J Am Acad Orthop Surg Glob Res Rev. 2025 Nov 10;9(11):e25.00079. doi: 10.5435/JAAOSGlobal-D-25-00079 (PMC12604657; doi:10.5435/JAAOSGlobal-D-25-00079)
Supplement: SUPPLEMENTARY MATERIAL [file jagrr-9-e25.00079-s004.docx]

JAAOS table 4

Supplemental Table 4: Estimated Effects of BMI & Other Predictors on Any Complications Following Total Joint Arthroplasty, Stratified by Joint

| **Independent Variable** | **Total Knee Arthroplasty** | | | | | **Total Hip Arthroplasty** | | | | |
| --- | --- | --- | --- | --- | --- | --- | --- | --- | --- | --- |
|  | ***Unadjusted*** | | | ***Adjusted (n = 406)*** | | ***Unadjusted*** | | | ***Adjusted (n = 198)*** | |
|  | **n** | **OR (95% CI)** | **p** | **OR (95% CI)** | **p** | **n** | **OR (95% CI)** | **p** | **OR (95% CI)** | **p** |
| BMI | 475 |  | 0.95† |  | 0.66† | 225 |  | 0.94† |  | 0.66† |
| > 50 |  | 0.99 (0.51, 1.95) | 0.98 | 0.81 (0.36, 1.82) | 0.60 |  | 1.18 (0.44, 3.20) | 0.74 | 0.63 (0.18, 2.25) | 0.47 |
| 45 – 49.99 |  | 0.92 (0.56, 1.53) | 0.76 | 0.78 (0.43, 1.39) | 0.39 |  | 0.99 (0.47, 2.05) | 0.97 | 1.18 (0.51, 2.73) | 0.70 |
| 40 – 44.99 |  | 1.00 (REF) | - | 1.00 (REF) | - |  | 1.00 (REF) | - | 1.00 (REF) | - |
| Sex | 475 |  |  |  |  | 225 |  |  |  |  |
| Female |  | 0.88 (0.54, 1.42) | 0.59 | 1.02 (0.58, 1.79) | 0.94 |  | 1.92 (1.00, 3.69) | 0.05* | 1.58 (0.76, 3.30) | 0.22 |
| Male |  | 1.00 (REF) | - | 1.00 (REF) | - |  | 1.00 (REF) | - | 1.00 (REF) | - |
| Race | 475 |  | 0.62† |  | 0.14† | 225 |  | 0.53† |  | 0.79† |
| Other |  | 0.87 (0.34, 2.24) | 0.78 | 3.21 (0.79, 13.12) | 0.10 |  | 0.32 (0.04, 2.87) | 0.31 | 0.57 (0.06, 5.69) | 0.63 |
| Black/African American |  | 0.77 (0.45, 1.31) | 0.33 | 0.71 (0.37, 1.37) | 0.31 |  | 0.80 (0.35, 1.82) | 0.60 | 0.79 (0.32, 1.96) | 0.61 |
| White |  | 1.00 (REF) | - | 1.00 (REF) | - |  | 1.00 (REF) | - | 1.00 (REF) | - |
| Ethnicity | 471 |  |  |  |  | 222 |  |  | - | - |
| Hispanic/Latino |  | 0.59 (0.27, 1.30) | 0.19 | 0.32 (0.09, 1.09) | 0.07 |  | 1.13 (0.30, 4.29) | 0.86 |  |  |
| Not Hispanic/Latino |  | 1.00 (REF) | - | 1.00 (REF) | - |  | 1.00 (REF) | - | 1.00 (REF) | - |
| Smoking | 475 |  | 0.50† |  | - | 225 |  | 0.35† |  | - |
| Current |  | 0.56 (0.21, 1.49) | 0.24 | - | - |  | 0.78 (0.25, 2.42) | 0.66 | - | - |
| Former |  | 0.98 (0.61, 1.60) | 0.95 | - | - |  | 1.53 (0.78, 2.99) | 0.21 | - | - |
| Never |  | 1.00 (REF) | - | 1.00 (REF) | - |  | 1.00 (REF) | - | 1.00 (REF) | - |
| Diabetes | 475 |  |  |  |  | 225 |  |  |  |  |
| Yes |  | 1.01 (0.64, 1.58) | 0.98 | - | - |  | 0.83 (0.43, 1.60) | 0.58 | - | - |
| No |  | 1.00 (REF) | - | 1.00 (REF) | - |  | 1.00 (REF) | - | 1.00 (REF) | - |
| Strong Anticoagulant Medication | 475 |  |  |  |  | 225 |  |  |  |  |
| Yes |  | 1.86 (1.19, 2.91) | 0.01* | 2.01 (1.19, 3.40) | 0.01* |  | 1.45 (0.78, 2.68) | 0.24 | 1.02 (0.48, 2.16) | 0.96 |
| No |  | 1.00 (REF) | - | 1.00 (REF) | - |  | 1.00 (REF) | - | 1.00 (REF) | - |
| Age at Surgery, 5-year increase | 475 | 0.97 (0.85, 1.10) | 0.60 | 0.89 (0.75, 1.06) | 0.18 | 225 | 1.00 (0.86, 1.16) | 0.98 | - | - |
| Charlson Comorbidity Index,  3-unit increase | 475 | 1.25 (0.98, 1.60) | 0.07 | 1.44 (1.05, 1.98) | 0.02* | 225 | 1.34 (0.98, 1.83) | 0.07 | 1.41 (0.98, 2.03) | 0.07 |
| Length of Surgery,  60-minute increase | 410 | 1.62 (1.15, 2.27) | 0.01* | 1.57 (1.07, 2.30) | 0.02* | 198 | 1.64 (1.03, 2.64) | 0.04* | 1.91 (1.11, 3.28) | 0.02* |

*Significant at α = 0.05 level

†Type 3 omnibus p-value for overall polytomous predictor effect

CI = Confidence Interval; OR = Odds Ratio
