# Supplementary material for: Analysis of the Resolution Rate of Complications in Obese Joint Replacement Patients
Source: J Am Acad Orthop Surg Glob Res Rev. 2025 Nov 10;9(11):e25.00079. doi: 10.5435/JAAOSGlobal-D-25-00079 (PMC12604657; doi:10.5435/JAAOSGlobal-D-25-00079)
Supplement: SUPPLEMENTARY MATERIAL [file jagrr-9-e25.00079-s005.docx]

JAAOS table 5

Supplemental Table 5: Complications Sorted by Type Perioperatively and During 90-Day Post-Operative Period

| DVT / Vascular Adverse Effect | 24 |
| --- | --- |
| Cardiac Adverse Effect | 13 |
| Infection (non-periprosthetic) | 44 |
| PJI / Hardware Loosening / Failure | 59 |
| Death | 1 |
| AKI | 5 |
| MSK / Neurological Adverse Effect | 35 |
| Fracture / Dislocation | 13 |
| CVA | 2 |
| Anesthesia Complication | 1 |
| URI | 4 |
| ABA* | 6 |
| PE | 4 |
| Lost to Follow Up | 4 |

DVT = deep vein thrombosis, PJI = prosthetic joint infection, AKI = acute kidney injury, MSK = musculoskeletal, CVA = cerebrovascular accident, URI = upper respiratory illness, acute blood loss anemia* = acute blood loss anemia requiring transfusion, PE = pulmonary embolism
